# The effect of a case-finding app on the detection rate of atrial fibrillation compared with opportunistic screening in primary care patients: protocol for a cluster randomized trial

**DOI:** 10.1186/s13063-021-05497-x

**Published:** 2021-08-09

**Authors:** Simon Gabriël Beerten, Tine Proesmans, Bert Vaes

**Affiliations:** 1 Department of Public Health and Primary Care, KU Leuven, Leuven, Belgium; 2 Qompium, Hasselt, Belgium

**Keywords:** Atrial fibrillation, Screening, FibriCheck®, Case finding

## Abstract

**Background:**

Atrial fibrillation is a cardiac arrhythmia commonly encountered in a primary care setting. Current screening is limited to pulse palpation and ECG confirmation when an irregular pulse is found. Paroxysmal atrial fibrillation will, however, still be difficult to pick up. With the advent of smartphones, screening could be more cost-efficient by making use of simple applications, lowering the need for intensive screening to discover (paroxysmal) atrial fibrillation.

**Methods/design:**

This cluster randomized trial will examine the effect of using a smartphone-based application such as FibriCheck® on the detection rate of atrial fibrillation in a Flemish general practice population. This study will be conducted in 22 primary care practices across the Flanders region of Belgium and will last 12 months. Patients above 65 years of age will be divided in control and intervention groups on the practice level. The control group will be subjected to standard opportunistic screening only, while the intervention group will be prescribed the FibriCheck® app on top of this opportunistic screening. The difference in detection rate between control and intervention groups will be calculated at the end of the study.

We will use the online platform INTEGO for pseudonymized data collection and analysis, and risk calculation.

**Discussion:**

Smartphone applications might offer a way to cost-effectively screen for (paroxysmal) atrial fibrillation in a primary care setting. This could open the door for the update of future screening guidelines.

**Trial registration:**

ClinicalTrials.gov NCT04545723. Registered on September 10, 2020.

## Administrative information

The order of the items has been modified to group similar items (see http://www.equator-network.org/reporting-guidelines/spirit-2013-statement-defining-standard-protocol-items-for-clinical-trials/).

**Table:** 

Title {1}	The effect of a case-finding app on the detection rate of atrial fibrillation compared with opportunistic screening in primary care patients: protocol for a cluster randomized trial
Trial registration {2a and 2b}.	Registered on clinicaltrials.gov on September 10, 2020 (NCT04545723).
Protocol version {3}	Version 2; April 29, 2020
Funding {4}	Funding requested.
Author details {5a}	**Simon Gabriël Beerten, MD, MSc** (corresponding author) Department of Public Health and Primary Care, KU Leuven, Leuven, Belgium simon.beerten@kuleuven.be **Tine Proesmans, MSc** Qompium, Hasselt, Belgium tine.proesmans@fibricheck.com **Bert Vaes, MD, PhD** Department of Public Health and Primary Care, KU Leuven, Leuven, Belgium bert.vaes@kuleuven.be
Name and contact information for the trial sponsor {5b}	Academic Centre of General Practice, KU Leuven Kapucijnenvoer 33 blok j – box 7001 3000 Leuven
Role of sponsor {5c}	Apart from the authors of this protocol, the sponsor is not involved in study design, data collection and analysis, interpretation, writing of the report or decision to submit for publication.

## Introduction

### Background and rationale {6a}

#### Incidence and prevalence of atrial fibrillation

Atrial fibrillation (AF) is one of the most common cardiac arrhythmias. Consequently, it is also frequently encountered in primary care. According to the INTEGO database (containing routinely collected general practice data of over 400,000 patients in Flanders [1]), the incidence of AF in a Flemish general practice population was 3.2 per 1000 patients in 2020 [2]. Estimates around the globe vary widely depending on the source. Current prevalence estimates range from 1 to 4% for Western countries [3], with a generally rising prevalence [4–6].

AF carries a significantly elevated risk of stroke. Relative risk of stroke in patients with AF varies between 2.6 and 4.5 for patients over 60 years old, compared to patients without AF [7]. The high morbidity and mortality of stroke is evident, and it is often the first presentation of silent AF [8]. It therefore seems useful to invest in screening strategies to identify those patients who have a higher risk of AF and need more intense monitoring, which may reduce healthcare costs brought about by hospitalizations for stroke [9, 10]. Furthermore, because some patients with AF will present with infrequent episodes—so-called paroxysmal atrial fibrillation (PAF)—the need for effective screening measures becomes clear [11]. For example, in a Portuguese population aged 40 and older, who were referred for 24-h continuous Holter monitoring, the prevalence of PAF was 2.5%, compared to 9.4% for persistent AF [12].

Still, many patients go undiagnosed. A recent study in the USA put the proportion of “silent” AF at 2.4% of the adult population [13].

#### Screening

Stroke risk in AF is commonly calculated using the CHA_2_DS_2_-VASc score (congestive heart failure, hypertension, age, diabetes, stroke, vascular disease, sex) [14]. Originally devised as a tool to predict the risk of stroke in AF, it may be less applicable to AF risk prediction in general [14]. Various other scoring systems have been developed over the years, and more recently the CHARGE-AF score has come to the foreground. For this score, data from three large cohorts in the US was used to predict AF risk in primary care settings [15].

The CHARGE-AF score is sufficiently validated to be used in AF stroke risk prediction and seems to perform better than the CHA_2_DS_2_-VASc score [16]. Furthermore, the CHARGE-AF score uses variables that are readily available from patient files in a primary care setting [15]. The variables of the (simple) CHARGE-AF score include age, race, height, weight, systolic and diastolic blood pressure, smoking status, antihypertensive medication use, diabetes, heart failure, and myocardial infarction [15].

Opinions on the best approach to screening for AF in general practice are not unanimous. There are various ways to go about it: pulse palpation, which, when revealing an irregular pulse, can lead to a clinical diagnosis of AF, or by performing an electrocardiogram (ECG) to confirm AF after palpation of an irregular pulse. These methods could be used in either specific at-risk populations, or generally in every patient above a certain age, a risk category on its own. Both methods are available in a primary care setting, but performing an ECG is especially time-consuming. The most cost-effective strategy at this moment is likely to involve opportunistically screening every patient 65 years and older by pulse palpation or heart auscultation [17], taking an ECG only in those who have an irregular pulse [18]. However, a systematic full screening of older patients is not cost-efficient [18, 19].

Patients who present with persistent AF are unlikely to be missed using the strategy outlined above. However, patients with the paroxysmal variant might still slip through and never be picked up using these methods. Holter registrations or even longer-term event recorders (in symptomatic patients) could partially solve this issue, but neither is very practical in general usage and their interpretation is very time-consuming.

#### Other options for screening

In recent years, various devices have been marketed as workarounds to solve the issue of detecting PAF in the general population. In addition, most offer a very user-friendly and easily accessible interface. Examples are heart rate and blood pressure monitors [20], one-lead ECG devices or smartphone apps [21, 22], or event recorder patches [23].

The global advent of smartphones might offer a unique opportunity to mediate the problem of detecting PAF [22, 24]. Apps carry the extra advantage of not needing additional hardware. The FibriCheck® app (Qompium, Hasselt, Belgium) has recently been developed to aid in the detection of PAF. It is based on the photoplethysmography technique, using only the phone’s built-in camera and flash [25]. The app has been shown to accurately detect AF in a primary care setting [26], with an estimated sensitivity and specificity of 95.6% and 96.6%, respectively. An ECG will still be necessary for diagnosis, but there will be less need for continuous ECG or event recording and the data can be easily visualized and interpreted by a general practitioner or cardiologist.

A recent pilot study by our team, to assess the ease-of-use and implementation of the FibriCheck® app in primary care, found high rates of patient satisfaction and compliance [27].

#### Aim of the study

Implementing the FibriCheck® app in a primary care setting could solve the problem of screening for AF and, specifically, detecting PAF. Therefore, the aim of this study is to investigate the effect of a case-finding strategy with the FibriCheck® application on the detection rate of AF in comparison with opportunistic screening (i.e. pulse palpation, followed by an ECG) in patients with a high risk of AF in general practice.

### Objectives {7}

The primary objective of this study will be to investigate the effect of a case-finding strategy with the FibriCheck® smartphone application on the detection rate of AF in comparison with opportunistic screening (i.e., pulse palpation, followed by an ECG) in patients with a high risk of AF in general practice. The secondary objective is to keep track of thrombo-embolic complications, death, and compliance with measurements.

### Trial design {8}

This study will be a cluster randomized trial: primary care practices will be randomized and divided into a control and intervention group. Allocation of control and intervention groups will be done by simple balanced randomization (1:1).

## Methods: Participants, interventions, and outcomes

### Study setting {9}

This study will take place in the eligible Flemish primary care practices in the INTEGO network.

The INTEGO network is a morbidity registry containing coded contents of the electronic health records of some 400,000 patients from 100 general practitioner’s offices all over Flanders. The information is automatically collected during daily practice and contains diagnoses, year of birth, gender, prescriptions, lab results, and various biomedical parameters such as blood pressure, height, weight, etc. [1]. The INTEGO procedures have been validated by the Belgian Privacy Commission. The registry is hosted on the Healthdata platform (www.healthdata.be) [28].

Patients coming to the eligible practices, as well as the practices themselves, will be recruited separately. They will fill in separate consent forms. After consent is given by the patient, the GPs of the recruited practices will explain the study outline and specific details, such as the installation and use of the app, at the first consultation, after which the study period for that specific patient will commence. General care will proceed as normal.

### Eligibility criteria {10}

To be eligible for inclusion in the study, *practices* must conform to the following conditions:

1. It is a Flemish primary care practice in the INTEGO network.
2. The practice uses an electronic health record (EHR), automatically linked to the INTEGO database.
3. ECG devices used for diagnosis (i.e., confirmation of AF) must be 12-lead.
4. The physician signs a specific study consent form.

To be eligible for inclusion in the control or intervention groups of the study, *patients* must conform to the following conditions:

1. The patient is 65 years or older.
2. The patient has an electronic medical record (EMR) in the practice. This EMR contains all the patient information, for instance regarding medical history and medication, and is managed by the general practitioner.
3. If the patient will be prescribed the FibriCheck® app, he/she signs the relevant patient consent form. Patients in the control group will also be required to sign a consent form.

Exclusion criteria for both the control and intervention group will be defined as follows:

1. The patient has already been diagnosed with AF.
2. The patient is already under anticoagulant therapy.
3. The patient has a pacemaker. Active pacing during measurements influences the results obtained with the FibriCheck® app [26].
4. The patient is unable to use the FibriCheck® application independently due to cognitive disorders, functional limitations, visual impairments… These will be identified using diagnosis codes in the patient file.

Drop-out criteria for patients in the intervention group will be defined as follows:

1. Less than 20 measurements with the FibriCheck® app during the study period.
2. Less than 2 measurements per day during the study period.
3. On patient request, for whatever reason.

### Who will take informed consent? {26a}

We will use two different consent forms in this study; practices will be given a study leaflet by the study personnel, outlining the study procedure and required data, together with their own consent form. They will be given one week to decide whether they want to participate. Patients in both arms of the study who want to be included will be given a study leaflet and a separate consent form, in this case by the participating physician. They will also be given one week to decide. They can make an appointment with their GP to hand in the signed consent form during this period. Patients who are not heard from will be contacted by email or phone to ask whether they still want to be included in the study and if so, to provide a signed consent form.

### Additional consent provisions for collection and use of participant data and biological specimens {26b}

Not applicable for this type of study, no biological specimens will be collected.

## Interventions

### Explanation for the choice of comparators {6b}

The most cost-effective strategy for AF screening at this moment is likely to involve opportunistically screening every patient 65 years and older by pulse palpation or heart auscultation [17], taking an ECG only in those who have an irregular pulse [18]. Systematic full screening of older patients is not cost-efficient [18, 19].

### Intervention description {11a}

In every cluster designated as an intervention group, patients aged 65 or older will be selected according to the criteria outlined above. Within this group, high-risk patients will be identified using the CHARGE-AF score and will be prescribed the FibriCheck® app. An integrated tool in the medical software package will calculate this score from the available parameters in the EHR. If needed, physicians will be asked to input missing data at the first consultation. Data for this score will be automatically extracted from the EHR from INTEGO. All high-risk patients will thus be flagged as such on the INTEGO platform. Physicians will also need to inform and educate patients about their high-risk status, as we consider this good clinical practice.

These high-risk patients will subsequently be informed about the study and possible enrolment and be given a study information letter describing the study procedure and purpose in detail.

Interested patients will be prescribed the FibriCheck® application after informed consent is given. Patients not in possession of a smartphone will be supplied one for the duration of the study. The application needs to be downloaded and patients will be asked to create a numbered account (in the format “patient00x,” etc.) and supply general details such as age and gender. A specific QR code links the patient’s account to the physician’s dashboard, so the latter can easily follow up.

The study will be conducted over the course of 12 months in general practices in the Flanders region of Belgium. Patients will be followed for a total of 4 weeks. Measurements and other data will be collected and interpreted at the end of this period. Positive measurements will be given immediate attention. A minimum of 20 measurements in total per patient in the intervention group is necessary to be considered for inclusion in the study.

To be able to track patients in the intervention group who were prescribed the FibriCheck® app, physicians will be asked to flag the specific patient file with a note saying “FC” or “FibriCheck,” or using the specific and personal ID created on the FibriCheck® app to link the app data to the patient file.

Every participant will be asked to measure at least twice a day (in the morning and in the evening) and if there are complaints such as dizziness or palpitations. After the measurement, the patient will be asked for his activities up to the measurement and certain symptoms. Participants are never asked to self-palpate their pulse, this will only take place at the GP’s office.

Measurements are available for the treating physician and a cardiologist, who will interpret the results within 24 h. Additionally, an electronic notification will be sent immediately to the interpreting physicians in case of an abnormal result. Physicians will be asked to review these results at least once every 24 h. At the end of every participant’s study period, a summarizing report will be sent to the treating physician.

When a positive result (red) has been found (indicating the possibility of AF), the patient will be contacted within 48 h for a formal 12-lead ECG. Patients themselves will also be instructed by the app to contact their GP for follow-up in case a possible AF has been found. If this ECG is inconclusive, a 2-week Holter measurement will be ordered. If AF is confirmed, rate or rhythm control and anticoagulant therapy will be started, or the patient will be referred to a cardiologist for further workup. The CHA_2_DS_2_-VASc score will be used to calculate the risk of thrombosis, and together with the HAS-BLED score [29], indicating the risk of bleeding, it will guide anticoagulant therapy [30].

If a positive FibriCheck® screening cannot be confirmed with 12-lead ECG or 2-week Holter, the patient files will be marked with a special code in the EHR and flagged for future, more intensive screening (with both the application and an ECG at consultation).

In addition to screening with the app, patients in the intervention group will also receive standard opportunistic screening: pulse palpation and a 12-lead ECG when an irregular rhythm is found.

#### Control group

In every cluster designated as a control group, patients aged 65 or older will be selected according to the criteria outlined above. The difference here is that patients will be given only the standard opportunistic screening. This is the current best practice [31].

### Criteria for discontinuing or modifying allocated interventions {11b}

A minimum of 20 measurements in total per patient in the intervention group is necessary to be considered for inclusion in the study. Patients will be expected to measure at least twice a day (morning and evening). The intervention will always be discontinued on patient request.

### Strategies to improve adherence to interventions {11c}

The FibriCheck® app will notify participants when to measure. Participants can always do more measurements, if they so desire.

### Relevant concomitant care permitted or prohibited during the trial {11d}

Apart from the FibriCheck® measurements, medical care will continue as usual for all participants.

### Provisions for post-trial care {30}

Not applicable: This is a purely screening-based trial; there are no adverse effects to be expected solely because of screening in the intervention group. It is possible that some patients might undergo unnecessary testing. However, we expect this number to be very low due to the high negative predictive value of the app, which was estimated to be 99.7% [26]. In addition, we expect the possible benefit of earlier detection of AF to outweigh this disadvantage.

### Outcomes {12}

#### Primary outcome measures

##### Detection rate of AF in patients 65 years and older

The detection rate of AF in both the control and intervention group will be calculated after 4 weeks. A significant difference in both groups will be noted. Compared with previous studies of similar design [32–34], we will realistically assume a 2-fold increase in the detection rate of AF in the intervention group to be significant.

##### Average time to detection of AF

We will compare the average time from the beginning of the study to the primary endpoint, the diagnosis of AF, in both study arms.

#### Secondary outcome measures

##### Thromboembolic complications

We will track the incidence of transient ischemic attack or ischemic stroke during the study period, in addition to the eventual difference between both study populations.

##### Death

We will keep track of all-cause mortality during the study period, as well as the difference in mortality between control and intervention populations.

##### Compliance

We will keep track of patient compliance during the study period (e.g., minimum number of measurements with FibriCheck®).

### Participant timeline {13}

The study will be conducted over the course of 12 months in general practices in the Flanders region of Belgium, planned to run from January 2022 to December 2022.

Allocation of control and intervention practices is planned to start around November 2021. Eligible participants will be followed in their usual primary care practice, and are recruited by the participating physician when they visit for whatever reason. They will be provided an information leaflet, a consent form, and 1 week decision time.

Participants will be followed for a total of 4 weeks. Measurements and other data will be collected and interpreted at the end of this period. After the total study period of 12 months, the resulting data will be analyzed.

**Fig. 1:**
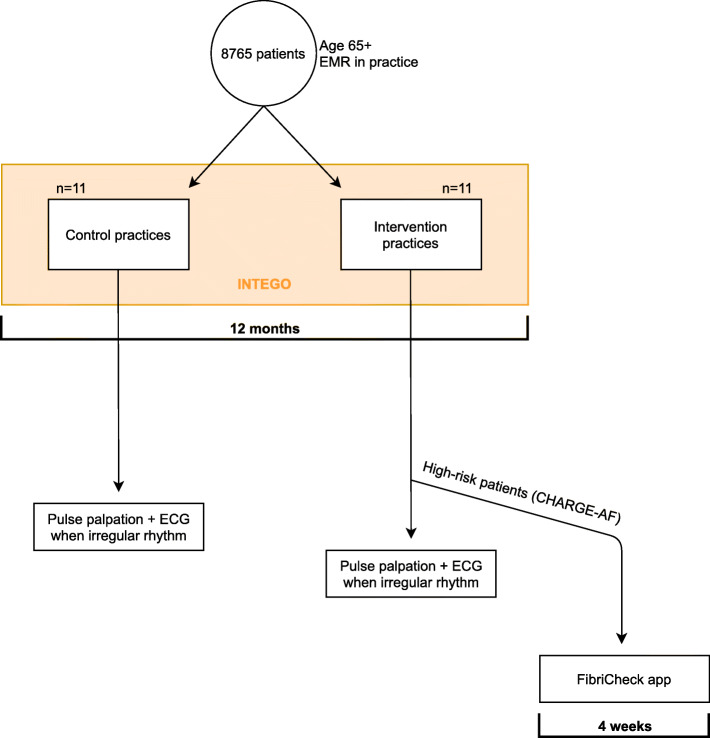
Visual representation of the study flow. EMR, electronic medical record

A schematic overview of the timeline can be found in Table 1. A visual representation of the study flow can be found in Fig. 1.

**Table 1:**
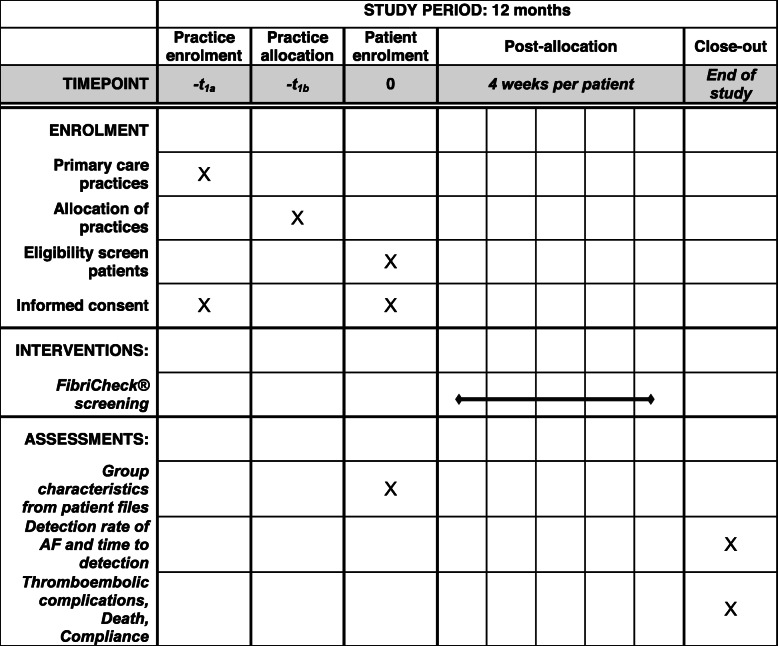
SPIRIT figure – Schedule of enrolment, interventions, and assessments.

### Sample size {14}

The calculation hereafter is similar to that of an earlier paper, comparable to ours [34]. We used an online calculator, which can be found at http://www.sample-size.net/sample-size-proportions.

The sample size calculation was performed for the primary outcome (AF detection rate at practice level). In the INTEGO database, we found a baseline AF incidence of 13.65/1000 patients, 65 years or older, in 2015. As stated above, we estimated a realistic absolute increase of 24.57 AF cases per 1000 patients per year in the intervention group. The power calculation, with a power of 0.80 and an α of 0.05, for a two-sided two-sample *t* test, with the incidence of AF per 1000 patients per year as the outcome variable, led to a sample size of 1835 patients in each arm of the study.

As we will view the results on the practice level, the number of practices needs to be determined, considering the intra-cluster correlation coefficient (ICC). A similar study found an ICC of 0.0027 (18). As we plan to let this study run for 12 months, we wish to include 400 patients (= *m*) per practice/physician. Calculating the variance inflation factor (1 + ICC (*m* – 1)) leads us to a value of 2.077. To accommodate the expected variance, we will have to include 1835 × 2.077, or 3811 patients per arm. We will assume a loss to follow-up of 15% [32]. This brings us to a total study population of 8765 patients, or approximately 11 control and 11 intervention practices.

### Recruitment {15}

Participants will be recruited as part of daily general practice, as outlined above. There are no additional strategies to ensure adequate recruitment.

## Assignment of interventions: allocation

### Sequence generation {16a}

Allocation of control and intervention groups will be done by simple balanced randomization (1:1), meaning that there will always be an equal number of control and intervention practices, assigned randomly. Enrolment of practices will be based on the in- and exclusion criteria described above and will be performed by researcher A, who is part of the trial. This will generate a numbered list of the eligible practices. Researcher B will then, independently and on a separate form, assign numbers (denoting a specific practice) to either the control or intervention groups by means of a computer-generated list of random numbers, i.e., simple randomization (for example, by using the website random.org to generate random numbers within a specified interval).

### Concealment mechanism {16b}

Researcher B will prepare sealed, opaque envelopes containing a paper that assigns a specific practice number to a study group, based on this randomly generated number sequence. The process of the envelope preparation up until sealing and storage in a locked compartment will be videotaped by researcher B, who will thereafter be excluded from every other aspect of the trial. Allocation papers should never be visible, only the envelopes and the numbers on them. The video will be stored on an external device, which will be put in the locked compartment.

After practice enrolment, researcher A will access the locked compartment with the envelopes and review the accompanying video to ensure proper envelope preparation. Without opening or tampering with the letters, researcher A will write the appropriate mailing address on the respective envelope, based on their numbered list of practices. The envelopes will only be opened by the practices if they have an unbroken seal. This protocol is adapted from Radford et al. [35].

### Implementation {16c}

All Flemish primary care practices in the INTEGO network conforming to the inclusion criteria will be contacted for inclusion in the study. Then, they will be randomly subdivided into intervention and control practices by the method described above. There will be an equal amount of intervention and control practices. In every practice, patients will be selected according to the previously defined in- and exclusion criteria, mentioned above. Later, high-risk patients in the intervention groups will be identified using the CHARGE-AF score, and will be prescribed the FibriCheck® app. High risk is defined as a 5-year risk of AF of at least 10% according to this score. The GPs in the intervention practices will explain the study outline to these patients and instruct them how to use the app, after which the study period begins for those patients.

## Assignment of interventions: Blinding

### Who will be blinded {17a}

Due to the nature of the study, it is not possible to blind at the practice level. Physicians will always be aware to which group they belong, as will the patients.

### Procedure for unblinding if needed {17b}

Not applicable: there is no blinding in this study.

## Data collection and management

### Plans for assessment and collection of outcomes {18a}

As stated above, to be able to track patients in the intervention group who were prescribed the FibriCheck® app, physicians will be asked to flag the specific patient file with a note saying “FC” or “FibriCheck,” or using the specific and personal ID created on the FibriCheck® app to link the app data to the patient file.

#### Data collected on demographics and clinical characteristics

To be able to compare both groups in terms of clinical similarity, we will provide relevant data that allows a reasonable comparison between the intervention and control groups. The data will be extracted from the medical software each practice uses by using the INTEGO procedures. The results will be analyzed and presented on the level of the practice. Baseline group characteristics relevant for the study will be assessed at the start of the study (Table 2).

**Table 2:** Collected data on group characteristics (for control and intervention groups)

Group characteristics (***n*** = 22)	
Mean age Gender distribution Mean body mass index Mean systolic/diastolic blood pressure, heart rate Smoking status (current, former, never) Antihypertensive medication Type II diabetes Heart failure Myocardial infarction	

If not already present in the patient file, missing data will be collected during this first interview. For example, if there is no mention of a patient’s blood pressure or height and weight, the participating physician will measure these parameters and put them into the EHR.

The general group characteristics schematized in Table 2 will be used to calculate the cardiovascular CHARGE-AF risk score of patients in the intervention group.

### Plans to promote participant retention and complete follow-up {18b}

There will be no specific strategies or plans to promote participant retention, in addition to those mentioned above.

### Data management {19}

The data gathered during the study of the patients that are prescribed the FibriCheck® app will be collected on the FibriCheck® platform, a cloud-based storage space. The research team (headed by the authors of this protocol) will be given credentials to access this platform and extract the data for interpretation and analysis (in the .csv (comma-separated values) file format), as will authorized employees of Qompium, the general practitioners of the selected practices and a cardiologist specifically assigned to the study. Only the research team will perform the extraction and analysis of these data. Measurements of the app are color-coded, indicating normal versus aberrant heart rhythm and their likelihood (from green over orange to red), or measurement errors (blue, indicating insufficient signal quality for analysis).

After collection, the data will be pseudonymized before access is granted to the FibriCheck® platform.

### Confidentiality {27}

The study data will be stored on a secured central server, accessible only for Qompium employees and the study researchers, including the participating GPs and cardiologists. The data will be accessible for the entire study period, and for 3 years thereafter, or longer if Belgian law so requires.

Personal information will be pseudonymized, as outlined above. Every participant will receive an individual and unique code, but names or identifiable information will not be collected to create this code.

Study data will be shared with Qompium as they provide the server on which the data will be stored. Data will not be shared with other third parties, national nor international.

### Plans for collection, laboratory evaluation, and storage of biological specimens for genetic or molecular analysis in this trial/future use {33}

Not applicable, no biological specimens will be collected in this study.

## Statistical methods

### Statistical methods for primary and secondary outcomes {20a}

We will analyze our data on an intention-to-screen basis, evaluating the outcomes on the practice (cluster) level. There will be no subgroup analyses.

The intention-to-screen analysis means we will include all participants of both the control and intervention groups in the final analysis, regardless of whether they did receive the stated screening intervention or dropped out before they could do so. This ensures that no bias is introduced by looking at the data on the practice level, for example by erroneously overestimating the effect of the intervention (screening with FibriCheck® on top of standard screening). This approach is valid as long as the studied population is appropriately randomized [36].

We will analyze the results on the cluster level, as the study is also randomized at the cluster level. Statistical efficiency should be sufficient with the cluster sizes being equal, as outlined above. As the unit of inference will be the cluster, and not the participant, we will apply standard *t* tests with inverse-variance weighting [37]. Given the fact that we have two independent study populations, we will use the paired (independent samples) *t* test. Because the observations within each cluster will likely be correlated, it is essential to account for this correlation, by choosing an appropriate method of analysis. The small number of clusters in this study could lead to an inflation of the type I error when applying individual level analyses. Inverse-variance weighting is one cluster-level strategy to reduce the type I error while maintaining sufficient statistical power [37].

### Interim analyses {21b}

There will be no interim analyses.

### Methods for additional analyses (e.g., subgroup analyses) {20b}

There will be no subgroup or any additional analyses.

### Methods in analysis to handle protocol non-adherence and any statistical methods to handle missing data {20c}

For sample size calculation, we already assumed a loss to follow-up (LTFU) of 15%. The barrier to entry in this study is very low and takes place in the context of a normal doctor’s appointment. Also, results will be read from the practice level, so all information will be collected in a coded fashion and patient confidentiality will be automatically ensured. This will also be outlined in the patient study information leaflet. We anticipate that these factors will reduce LTFU as much as possible.

We will keep track of LTFU—for any reason—during the study and compare LTFU between control and intervention group at the end. Any significant difference will be discussed. Patients who do not complete the 4-week study period (for whatever reason), will not be included in the results.

For missing data, we will use multiple imputation to obtain complete datasets.

### Plans to give access to the full protocol, participant level data, and statistical code {31c}

The full dataset will be available from the corresponding author on reasonable request after the trial is finished.

## Oversight and monitoring

### Composition of the coordinating center and trial steering committee {5d}

The study data will be stored on a server provided by Qompium, the firm that also developed the FibriCheck® application. TP is an employee of Qompium and will be directly involved in the data collection and analysis. Other employees of Qompium are not directly involved in the study, data collection or analysis. They can always give assistance in case of technical issues. There is no coordinating center or steering committee.

### Composition of the data monitoring committee, its role, and reporting structure {21a}

As this is a screening trial with no expected harms purely as a result of the intervention, a data monitoring committee is deemed not necessary.

### Adverse event reporting and harms {22}

This is a purely screening-based trial; there are no adverse effects to be expected solely because of screening in the intervention group.

### Frequency and plans for auditing trial conduct {23}

There will be no trial conduct auditing.

### Plans for communicating important protocol amendments to relevant parties (e.g., trial participants, ethical committees) {25}

Changes to the study protocol will be directly communicated to the Ethics Committee of University Hospitals Leuven, and the participating physicians. Any relevant modifications in the study protocol for patients already included in the study will be communicated individually.

Patients yet to be recruited will receive a modified version of the protocol before giving informed consent.

### Dissemination plans {31a}

The results of the trial will be published in the scientific literature, as well as included in trial databases.

## Discussion

To our knowledge, this will be the first study to evaluate the influence of a stand-alone smartphone application on the detection rate of AF compared to a control group. There are certain indispensable elements on which the study will hinge. Certain issues can be expected here.

Most importantly, the availability of correctly coded data is essential for the study to be conducted. Physicians must code diligently and completely. Without coded data and diagnoses, this study will be difficult to perform on a large basis. In the INTEGO database, it is possible to identify practices who code well, based on frequency and amount of coded diagnoses per consultation.

To screen high-risk patients, we will use the sufficiently validated CHARGE-AF score. Certain EMD software packages, however, do not allow custom searches based on every parameter in this score. Ideally, a tool could be developed that automatically calculates the CHARGE-AF score across platforms for every individual patient and notifies the physician if a patient needs to be screened.

The 4-week screening period was determined from other studies [34, 38]. A similar study found an increase in the cumulative diagnostic yield for AF from 0.4% for a single measurement to 1.4% for a week-long screening [38]. Based on these results, we believe that a screening period of 4 weeks will provide sufficient diagnostic yield.

There are certain limitations to this study as well. For the data to be correctly sent to the cloud, patients must have a working internet connection. Also, patient compliance is important. This will have an impact on the detection rate of AF that we will find in both study groups.

A recently published study of similar design found no increased diagnostic yield for AF when comparing opportunistic screening with regular care [39]. This study differs from ours in some important aspects, most notably the fact that opportunistic screening was limited to GP practices, whereas in our study the screening is extended with the use of a smartphone application.

There have also been a number of other studies regarding community-wide screening for AF using smartphone apps or mobile devices. A study done in Hong Kong, for example, used the smartphone-based AliveCor device for community screening [22]. The device was used by study personnel on community-recruited volunteers, and a high diagnostic yield was found. The main differences with our study were the fact that there was selection bias due to community recruitment of volunteers, and that medical history was self-reported. There was also no standard twelve-lead ECG taken as a reference.

Another British study using the same device has more similarities to our study protocol, such as using primary care records and self-administered screening by the participants [31]. Here, patients in the intervention arm were asked to measure twice weekly with the AliveCor device. By this method, a fourfold increase in the detection rate of AF was found.

## Trial status

This study protocol (version 2; dated April 29, 2020) with registration number B3222020000036 was approved by the Ethics Committee of University Hospitals Leuven, Belgium on May 14, 2020.

Recruitment has not yet started. It is planned to begin at the end of 2021.

## Abbreviations

AF: Atrial fibrillation
ECG: Electrocardiogram
EHR: Electronic health record
EMR: Electronic medical record
ICC: Intra-cluster correlation coefficient
LTFU: Loss to follow-up
PAF: Paroxysmal atrial fibrillation
